# Interaction of Local Anesthetics with Biomembranes Consisting of Phospholipids and Cholesterol: Mechanistic and Clinical Implications for Anesthetic and Cardiotoxic Effects

**DOI:** 10.1155/2013/297141

**Published:** 2013-09-23

**Authors:** Hironori Tsuchiya, Maki Mizogami

**Affiliations:** ^1^Department of Dental Basic Education, Asahi University School of Dentistry, 1851 Hozumi, Mizuho, Gifu 501-0296, Japan; ^2^Department of Anesthesiology and Reanimatology, University of Fukui Faculty of Medical Sciences, 23-3 Matsuokashimoaizuki, Eiheiji-cho, Yoshida-gun, Fukui 910-1193, Japan

## Abstract

Despite a long history in medical and dental application, the molecular mechanism and precise site of action are still arguable for local anesthetics. Their effects are considered to be induced by acting on functional proteins, on membrane lipids, or on both. Local anesthetics primarily interact with sodium channels embedded in cell membranes to reduce the excitability of nerve cells and cardiomyocytes or produce a malfunction of the cardiovascular system. However, the membrane protein-interacting theory cannot explain all of the pharmacological and toxicological features of local anesthetics. The administered drug molecules must diffuse through the lipid barriers of nerve sheaths and penetrate into or across the lipid bilayers of cell membranes to reach the acting site on transmembrane proteins. Amphiphilic local anesthetics interact hydrophobically and electrostatically with lipid bilayers and modify their physicochemical property, with the direct inhibition of membrane functions, and with the resultant alteration of the membrane lipid environments surrounding transmembrane proteins and the subsequent protein conformational change, leading to the inhibition of channel functions. We review recent studies on the interaction of local anesthetics with biomembranes consisting of phospholipids and cholesterol. Understanding the membrane interactivity of local anesthetics would provide novel insights into their anesthetic and cardiotoxic effects.

## 1. Introduction

**Figure 1:**
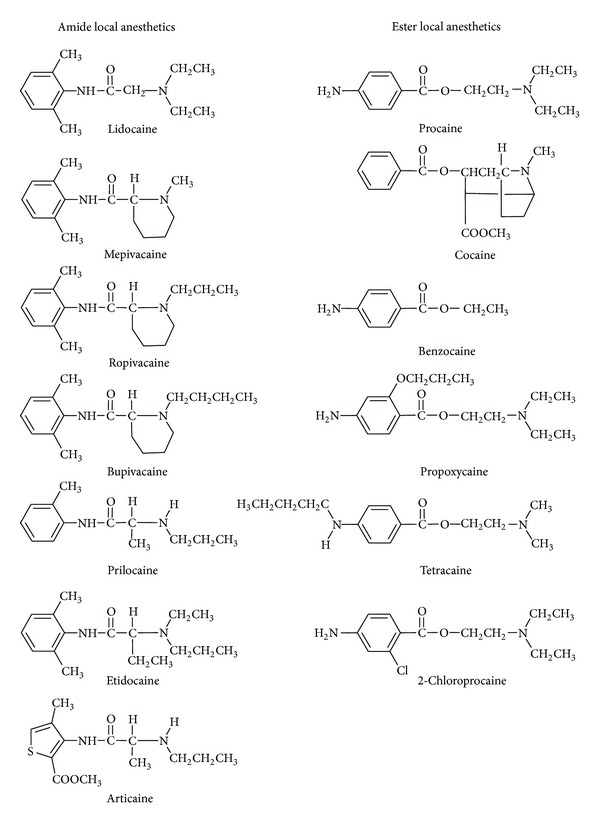
Representative amide and ester local anesthetics.

Local anesthetics clinically used so far have the common chemical structure that is composed of three portions: the hydrophobic moiety consisting of an aromatic ring, the intermediate chain, and the hydrophilic moiety consisting of an amino terminus. The aromatic residue confers lipid solubility on a drug molecule, whereas the ionizable amino group confers, water solubility. The intermediate portion provides the spatial separation between hydrophobic and hydrophilic end and structurally classifies local anesthetics into amide type and ester type (Figure 1).

**Figure 2:**
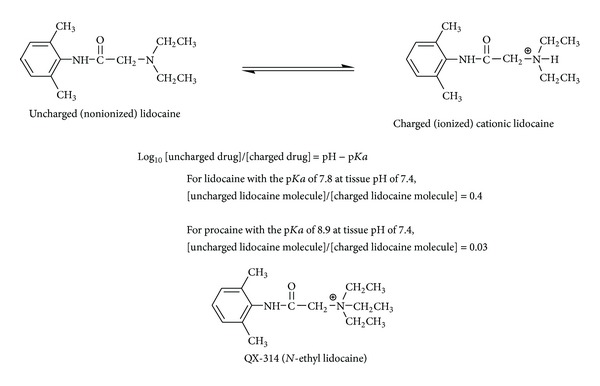
Uncharged and charged local anesthetics and permanently charged derivative.

Because of the presence of substituted amino groups, local anesthetics are referred to as the bases with p*K*a values ranging from 7.7 to 8.1 at 37°C for the amide type and from 8.4 to 8.9 at 37°C for the ester type [1], so they exist in uncharged and positively charged form. After injected, local anesthetics show an in vivo equilibrium between the uncharged and the charged fraction of molecules. According to the Henderson-Hasselbalch equation, the percentage of uncharged molecules depends on the p*K*a and medium pH (Figure 2). The pharmacokinetics and the mode of action of local anesthetics are closely related to their interaction with membrane lipids. Uncharged molecules can predominantly diffuse through the lipid barriers of nerve sheaths and penetrate into and across the lipid bilayers of cell membranes to reach the acting sites. The pH-dependent effects of local anesthetics have been discussed in association with the pH changes in inflammation, ischemia, and several diseases [2–4].

It is generally recognized that local anesthetics primarily affect sodium channels in sensory nerve fibers and cardiomyocytes [5]. Blocking peripheral nerves to induce local anesthesia is achieved at the relatively high concentrations of drugs in local areas. At lower concentrations, certain local anesthetics also show more subtle effects on hearts, which are clinically important as an antiarrhythmic agent. In addition, lidocaine administered systemically may be useful for reducing the excitability of nociceptive sensory neurons [6].

**Figure 3:**
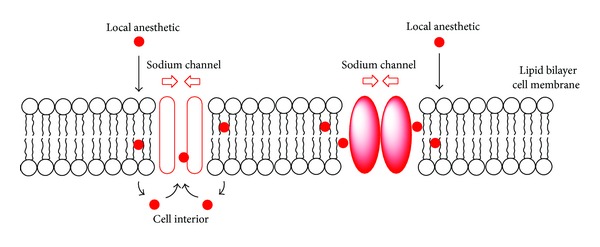
Channel protein-interacting and membrane lipid-interacting local anesthetics.

Local anesthetics block the conduction of nerve impulses by affecting the influx of ions through transmembrane channels. Proposed mechanistic theories include the action on membrane-embedded ion channels, membrane-associated enzymes, membrane lipids, and water. In the protein-interacting theory, local anesthetics are presumed to inhibit the generation and conduction of action potentials in nerve cells and cardiomyocytes by binding to the intracellular site of voltage-gated sodium channels embedded in membrane lipid bilayers (Figure 3). Local anesthetics specifically bind to the D4-S6 region of the *α*-subunit of neural sodium channels. Since this site is intracellular or cell interior, drug molecules are absolutely required to be in uncharged hydrophobic form for accessing there.

Drugs acting on proteinous channels, receptors, and enzymes also have the ability to act on lipid bilayers and modify membrane physicochemical properties [7]. Local anesthetics interact with membrane lipids to change fluidity, order, microviscosity, and permeability of membranes [8] and also influence the electrostatic potential across lipid bilayers, which may affect the functions of voltage-gated ion channels [7, 9]. They are presumed to act on lipid bilayers, with the resultant alteration of the membrane lipid environments surrounding transmembrane proteins and the subsequent change of protein conformation, thereby influencing the channel activity (Figure 3). Considering the diversity in their chemical structures, a specific single acting site within ion channels has been suggested to be unlikely for all of local anesthetics [10]. Local anesthetics affect various functional membrane proteins such as potassium ion channel, calcium ion channel, acetylcholine receptor, adrenergic receptor, GABA_A_ receptor, *γ*-aminobutyric acid receptor, glycine receptor, adenylate cyclase, phospholipase A_2_, and Na,K-ATPase [11–13]. Such broad spectra are interpretable by the fundamental action on a target common to local anesthetics, membrane lipids [14]. A wide range of pharmacological and toxicological properties of local anesthetics cannot be ascribed to binding to a single protein target alone [7], so it is reasonable to assume that the interaction of local anesthetics with lipid bilayer biomembranes contributes, at least partly but significantly, to their anesthetic and toxic effects. It has been recently suggested that the responsibility of membrane lipids for and the role of specific membrane component(s) in anesthetic mechanisms should be reverified [15].

## 2. Drug and Membrane Interaction

### 2.1. Membrane Preparation

Because biological membranes are a very complex system, biomimetic model membranes consisting of lipids have been employed more frequently than natural biomembranes for studying the drug and membrane interaction [16]. The advantages of using protein-free lipid membranes are that one can easily manipulate the reaction condition and the membrane lipid composition, focus only on the interaction between drugs and membrane lipids, avoid the interference from other membrane components, and quantitatively evaluate the membrane response to drugs. Model membranes with the lipid bilayer structure can be prepared to mimic cellular and plasma membranes of interest by adjusting their lipid compositions. One of biomimetic model membranes widely used is a liposome, the vesicle with concentric lipid layers in which an aqueous volume is entirely enclosed by membranous lipids. A unilamellar vesicle is characterized by a single lipid bilayer consisting of inner and outer leaflet.

**Figure 4:**
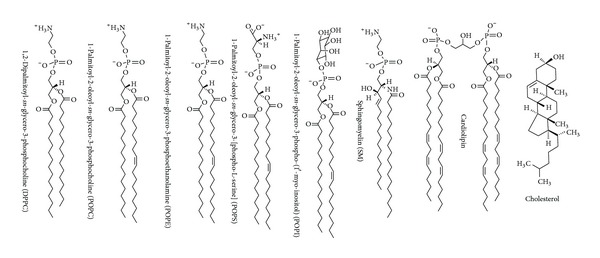
Membrane lipid components.

The major phospholipids constituting biological membranes are glycerophospholipids with the content of 40–60 mol% in total lipid fraction. They consist of a glycerol backbone on which two fatty acids are commonly esterified at the stereospecifically numbered *sn*-1 and *sn*-2 position. The third carbon atom of a glycerol backbone supports the polar head group composed of choline, ethanolamine, serine, inositol, and so forth, which are linked to a negatively charged phosphate group. Representative lipids used for preparing biomimetic membranes are shown in Figure 4. The most abundant steroid in biological membranes is cholesterol that comprises four fused cycles in the *trans*-configuration, a hydroxyl group at the 3-position, a double bond between the carbon 5 and 6, and an iso-octyl lateral chain at the 17-position. Liposomal membranes are prepared by the aqueous dispersion of these authentic lipids (either a single component or a mixture of several lipids) or lipids extracted from cells.

### 2.2. Membrane Interactivity Analysis

The drug and membrane interaction can be analyzed by different techniques such as electron spin resonance, differential scanning calorimetry, nuclear magnetic resonance, X-ray diffraction, and fluorometry. Of spectroscopic and biophysical methods, fluorescence polarization has been most frequently used to determine the change in membrane fluidity, which is usable as an index for quantitatively evaluating the membrane interactivity of drugs. The term “membrane fluidity” may mean a combination of different kinds of membrane component mobility like the flexibility of membrane phospholipid acyl chains, the lateral or transverse diffusion of molecules in lipid bilayers, and the membrane lipid phase transition.

The polarization of fluorescence emitted by a membrane-incorporated fluorophore reflects its mobility in the surrounding membrane lipid environments. Fluorescence polarization is measured by excitation performed with monochromatic light that is vertically polarized and the emission intensity detected through an analyzer oriented parallel or perpendicular to the direction of polarization of the excitation light. A variety of fluorophores such as 1,6-diphenyl-1,3,5-hexatriene (DPH), 1-(4-trimethylammoniumphenyl)-6-phenyl-1,3,5-hexatriene (TMA-DPH), *N*-phenyl-1-naphthylamine (PNA), and *n*-(9-anthroyloxy)stearic acid (*n* = 2, 6, 9 and 12, *n*-AS) are usable for measuring fluorescence polarization. These probes penetrate into membranes to align with phospholipid acyl chains and locate in different membrane regions based on their chemical structures and lipid-interacting properties, indicating the fluidity of the membrane region specific to each individual probe. They are subject to the rotational restriction imparted by lipid bilayer rigidity or order. Drugs interact with lipid bilayers to produce more fluid (less rigid) or disordered membranes, which facilitate the probe rotation to emit the absorbed light in all directions, resulting in a decrease in fluorescence polarization. On the contrary, more rigid (less fluid) or ordered membranes produced by drugs disturb the probe rotation to emit the absorbed light in all directions, resulting in an increase in fluorescence polarization. Compared with controls, decreased and increased polarization means an increase of membrane fluidity (membrane fluidization) and a decrease in membrane fluidity (membrane rigidification), respectively.

## 3. Local Anesthetic Membrane Interaction

Yun et al. [17] investigated the effects of lidocaine, bupivacaine, prilocaine, and procaine on synaptosomes isolated from bovine cerebral cortex and liposomes prepared with extracted lipids by measuring the fluorescence polarization with 2-AS and 12-AS. They showed that these local anesthetics increased the membrane fluidity by preferentially acting on the hydrocarbon interior. Tsuchiya et al. [18, 19] reported that lidocaine, prilocaine, bupivacaine, ropivacaine, and mepivacaine interacted with liposomal membranes to increase the membrane fluidity by measuring DPH, TMA-DPH, and PNA fluorescence polarization. They also revealed that the interactivity with lipid bilayer membranes is to a great extent consistent with the local anesthetic potency [20]. Local anesthetics are speculated to interact with lipid bilayers to rearrange the intermolecular hydrogen-bonded network among phospholipid molecules in association with the liberation of hydrated water molecules on membranes and also alter the orientation of the P–N dipole of phospholipid molecules, resulting in an increase in membrane fluidity [17]. However, the membrane lipid-interacting theory has been criticized as to whether local anesthetics can affect biomembranes at clinically relevant concentrations, whether local anesthetic molecules in charged form can interact with lipid bilayers, and whether local anesthetics like bupivacaine and ropivacaine can stereostructure-specifically act on lipid membranes to show the potency different between stereoisomers. These subjects are discussed in Sections 4, 5, 6, and 8.

## 4. Membrane Interaction at Clinically Relevant Concentrations

The concentrations of lidocaine typically used in the clinical setting for dentistry are 1–3% (w/v) [14]. At 0.03–0.08 times these concentrations, Tsuchiya and Mizogami [20] applied lidocaine to liposomal membranes consisting of 100 mol% DPPC or POPC and found that lidocaine increases the membrane fluidity by determining DPH fluorescence polarization decreases. At 0.03–0.25 times lower concentrations of usually administered 0.125–0.75% (w/v) [21], Mizogami et al. [19, 20] revealed that bupivacaine and ropivacaine interact with nerve cell-mimetic membranes to increase the membrane fluidity, resulting in decreases in DPH, PNA, and TMA-DPH fluorescence polarization. The interaction of local anesthetics with nerve cell membranes at clinically relevant concentrations is supported by the fact that membrane-interactive agents including local anesthetics are more effective in modifying the physicochemical property of biological membranes than artificial model membranes [17].

Önyüksel et al. [22] reported that bupivacaine enhanced the carboxyfluorescein release from liposomes with increasing the cardiolipin content in liposomal membranes. Tsuchiya et al. [23] found that bupivacaine increases the fluidity of 2.5–12.5 mol% cardiolipin-containing membranes to decrease DPH fluorescence polarization at 10–50 *μ*M, which corresponded to the blood concentrations for free bupivacaine to produce the cardiac collapse and depress the myocardial function. When dogs were administered with bupivacaine of the cumulative dose of 21.7 ± 2.6 mg/kg, the mean plasma concentration of free and total bupivacaine at cardiovascular collapse was 20 and 63 *μ*M, respectively [24]. The dose of bupivacaine of 7.2–8.8 mg/kg caused the arrhythmias of dogs, in which the mean concentration of free and total bupivacaine in plasma reached 4 and 17 *µ*M, respectively [25]. In the range of these cardiotoxic concentrations, bupivacaine is able to interact with cardiolipin-containing biomembranes and significantly change the membrane fluidity.

The membrane effects were frequently investigated by measuring fluorescence polarization with DPH in previous studies [19, 20, 23]. The degrees of local anesthetics-induced changes in DPH polarization correspond to the functional changes of lipid bilayer membranes and membrane-embedded proteins [23, 26, 27], possibly producing the pharmacologic and toxic effects of local anesthetics.

Membrane-acting drugs accumulate in lipid bilayers and their local membrane concentrations are much higher than their concentrations in the bulk aqueous phase to show the intramembrane concentrations being hundreds of times higher than the aqueous concentrations [28]. Their concentrations in lipid bilayer membranes are consistent with the properties of local anesthetics to interact with biomembranes and modify the membrane fluidity at clinically relevant concentrations. While general anesthetics interact with biomembranes as well as local anesthetics do, they show the water-membrane interfacial concentrations much higher than the concentrations in bulk hexane [29]. Kopeć et al. [7] speculated that the drug dosage necessary to effect a protein-mediated response may be higher than that needed for a membrane-mediated mechanism, so clinical dosages for targeting membranes may be lower. They also suggested that the drug membrane concentration is few orders of magnitude higher than typically found in plasma.

## 5. Membrane Interactivity Depending on Lipid Components

### 5.1. Lidocaine Derivative QX-314

One reason the membrane lipid-interacting theory is controversial is the general recognition that the pharmacological activity of charged drugs is negligible when they are applied extracellularly. QX-314 (*N*-(2,6-dimethylphenylcarbamoylmethyl)triethylammonium chloride, *N*-ethyl lidocaine) is a quaternary derivative of lidocaine whose structural difference is only an *N*-ethyl group but renders its parent structure permanently charged and water soluble (see Figure 2 for QX-314 structure). In contrast to lidocaine, this cationic agent shows only very slow penetration into membrane lipid bilayers and cannot readily cross lipid barriers [30]. In previous experiments, extracellularly applied QX-314 was not effective in blocking action potentials, whereas the intracellular application of QX-314 exerted the significant effects. Surprisingly, however, Lim et al. [31] recently reported that extracellularly applied QX-314 produced the long-lasting local anesthesia with a slow onset in guinea pigs and mice, challenging the conventional notion that charged molecular species are inactive when administered from the external side of cells [32].

Tsuchiya and Mizogami [20] compared the interactivity of lidocaine and QX-314 with biomimetic model membranes by measuring DPH fluorescence polarization. Lidocaine increased the membrane fluidity of liposomes consisting of 100 mol% DPPC and POPC, but not QX-314. These comparative results agree with previous reports that charged local anesthetics were ineffective on cell membranes when applied extracellularly [32]. On the other hand, QX-314 was revealed to decrease the DPH polarization values of both nerve cell-mimetic membranes and liposomal membranes consisting of POPS, 1-palmitoyl-2-oleoyl-*sn*-glycero-3-phosphate (POPA), 1-palmitoyl-2-oleoyl-*sn*-glycero-3-[phospho-*rac*-(1-glycerol)] (POPG), or cardiolipin and to show almost the same membrane-fluidizing effect as lidocaine [20]. Membrane lipid components and their compositions are very likely to determine whether the charged molecules of local anesthetics can modify membrane physicochemical properties or not.

### 5.2. Interaction with Specific Phospholipids

A zwitterion is a neutral molecule with positive and negative electrical charge within the molecule. The major membrane phospholipids are zwitterions with the polar head groups consisting of anionic phosphate and cationic quaternary ammonium centers (Figure 4). When comparing phospholipid species, QX-314 was not effective in changing the fluidity of biomimetic membranes consisting of zwitterionic phospholipids such as DPPC, POPC, and POPE [20]. POPS has an additional carboxyl group, neither POPA nor POPC has an ammonium group, and cardiolipin has two phosphate groups. QX-314 significantly decreased the DPH polarization values of biomimetic membranes containing these anionic phospholipids to produce the membrane fluidization as well as lidocaine [20]. Amphiphilic drugs like local anesthetics cause not only the hydrophobic interaction with phospholipid aliphatic acyl chains but also the electrostatic interaction with phospholipid polar head groups. The difference between anionic and zwitterionic phospholipid membranes suggests the electrostatic interaction between positively charged anesthetic molecules and negatively chargeable phospholipid head groups. The interaction of local anesthetics with specific membrane phospholipids also provides a mechanistic clue to explain their structure-dependent cardiotoxicity as described in Section 7.2.

## 6. Change of Membrane Interactivity

### 6.1. Anesthetic Efficacy Reduced by Inflammation

Inflammatory diseases alter the pharmacodynamics and pharmacokinetics of various drugs, resulting in a decrease in their beneficial effects and/or an increase in their adverse effects [33, 34]. Such an alteration by inflammation is well known in clinical dentistry, where the local anesthetic failure or the difficulty to obtain satisfactory analgesia commonly occurs in the situations of pulpitis and apical periodontitis [2]. The anesthetic efficacy of lidocaine, mepivacaine, and articaine injections is remarkably reduced in the teeth with pulpitis [35, 36]. The presence of inflammation in dental pulp was reported to cause the inferior alveolar nerve block to fail in approximately 30–45% of cases [37].

### 6.2. Verification of Conventional Acidosis Theory

Acidic metabolites like lactic acid are increasingly produced and concentrated in inflamed tissues, thereby inducing the acidosis that lowers the tissue pH at least the order of 0.5–1.0 pH unit [38, 39]. The p*K*a values of almost all of local anesthetics in dental use are larger than 7.5 at 37°C [1], so a greater proportion of administered drugs exist as positively charged molecules in and near inflammatory lesions. Because cationic molecules are much less in membrane permeability and membrane interactivity under acidic conditions, the local anesthetic effects should be remarkably decreased, leading to the mechanistic theory that the local anesthetic efficacy is reduced by inflammatory tissue acidosis. This acidosis theory has been conventionally accepted due to its theoretical simplicity and understandability and most frequently cited for explaining the reduction of local anesthetic efficacy. However, even the fundamental subject as to whether local anesthetics decreasingly interact with lipid bilayers under acidic conditions has not been experimentally confirmed.

Tsuchiya et al. [40, 41] compared the membrane effects of local anesthetics with lowering the reaction pH and varying the composition of membrane lipids. When liposomal membranes consisting of 100 mol% DPPC were treated at pH 5.9–7.4 with lidocaine, prilocaine, and bupivacaine of 0.05–0.2% (w/v), all of the tested local anesthetics increased the membrane fluidity in 15 minutes almost corresponding to their onset time. Compared with pH 7.4, however, their membrane effects were significantly decreased at pH 6.4 that is almost comparable to inflamed tissue conditions [38]. Such a pH dependence in DPPC membranes supports the acidosis theory.

On the other hand, lidocaine, prilocaine, and bupivacaine of 0.05–0.2% (w/v) were revealed to interact even at pH 6.4 with nerve cell-mimetic membranes prepared with POPC, POPE, POPS, SM, and cholesterol, resulting in a significant increase in the membrane fluidity [40, 41]. Their membrane-fluidizing effects under acidic conditions are in conflict with the conventional acidosis theory. Local anesthetics showed less fluidizing effects at pH 6.4 on liposomal membranes consisting of DPPC, POPC, POPE and SM, whereas their effects on POPS liposomal membranes were significantly greater at pH 6.4, contradicting the relative decrease of uncharged molecules, but correlating to the relative increase of charged molecules. Phospholipid DPPC, POPC, POPE, and SM are zwitterionic, but POPS is acidic. Under acidic conditions, the cationic moieties of local anesthetics are likely to electrostatically interact with the anionic head groups of acidic phospholipids, resulting in the modification of membrane fluidity. Therefore, the tissue acidosis is not necessarily related to the local anesthetic efficacy reduced by inflammation.

Punnia-Moorthy [38] revealed that inflamed tissues lower only the order of 0.5 pH unit. In animal experiments, the tissues were found to rapidly buffer the excess acidity after acidic solution infiltrations and such a pH-buffering ability was more potent in inflamed tissues [42]. These pathophysiological features are also unfavorable for the conventional mechanism based on tissue acidosis.

### 6.3. Possible Mechanism Alternative to Acidosis

Peroxynitrite has been implicated in the pathogenesis of various diseases including inflammation. Inflammatory cells produce peroxynitrite by the reaction between nitric oxide and superoxide anion, both of which are present in inflamed tissues. Peroxynitrite is also known to react with lidocaine and bupivacaine [43, 44]. Ueno et al. [41] focused on inflammatory peroxynitrite responsible for the local anesthetic failure of inflamed tissues. They treated nerve cell-mimetic membranes with lidocaine, prilocaine, and bupivacaine of 0.05–0.2% (w/v) together with 50 *μ*M peroxynitrite at pH 7.4 and 6.4. The following DPH fluorescence polarization measurements showed that the membrane-fluidizing effects of local anesthetics were inhibited by the peroxynitrite treatment. Such inhibitions are causable by peroxynitrite directly acting on anesthetic molecules [45] and indirectly on membrane lipids [46].

Since local anesthetic solutions are injected relatively near to acutely inflamed tissues in dental anesthesia [14], peroxynitrite could interfere with the maxillary and mandibular nerve block by local anesthetics. Inflammation possibly produces hyperexcitability or hyperalgesia [47, 48]. The absorption of local anesthetics into the circulatory system to remove them from the administered site is promoted in inflammatory lesions [42]. In addition to these pathophysiological changes, inflammatory peroxynitrite is responsible for reducing the efficacy of topical and infiltration anesthesia.

## 7. Membrane Interaction Associated with Cardiotoxicity

### 7.1. Adverse Effects of Local Anesthetics

The administration of local anesthetics is accompanied by the potential risk that is rare but could be fatal complication. The adverse effects of local anesthetics include systemic and local toxic reactions and allergic reactions mostly related to ester-type drugs. The systemic toxicity occurs in the cardiovascular and central nervous system, while the local toxicity may lead to neurotoxicity, transient neurological symptom, or myotoxicity [49]. The cardiovascular system toxicity of commonly used amide local anesthetics has been clinically of much interest since the report of cardiotoxic effects of bupivacaine and etidocaine [50].

Local anesthetics, especially bupivacaine, cause cardiac disorders that consist of the initial depression of intraventricular conduction followed by reentrant arrhythmia. Great concerns over their cardiotoxicity include profound bradycardia, arrhythmia, myocardial depression, and eventually cardiovascular collapse, which are induced when the drug concentrations in blood are elevated by an accidental intravenous injection and an absolute overdose. Although bupivacaine has been widely used for cutaneous infiltration, regional nerve block, epidural anesthesia, and spinal anesthesia in surgery and obstetrics, this long-acting agent is greater in toxicity compared with shorter-acting amide local anesthetics. The rank order of cardiotoxic potency has been estimated to be bupivacaine > ropivacaine > lidocaine > prilocaine [51]. However, the detailed mechanism(s) for structure-specific or structure-dependent cardiotoxicity has not been clear.

### 7.2. Interaction with Membrane Cardiolipin

Although myocardial ion channels are referred to as the primary target of local anesthetics, another site of toxic action is assumed to contribute to the cardiotoxicity discrimination between structurally different drugs. Besides binding to ion channels, local anesthetics act on membrane lipids to modify permeability, fluidity, lipid packing order, and lipid phase transition of biomembranes [22, 23, 52]. The inhibitory effects of anesthetic agents on ionic current generations also require the molecular interplay of ion channel proteins and membrane lipids [53].

As cardiotoxic drugs affect the permeability of mitochondrial membranes, local anesthetics also act on lipid bilayers and increase the membrane permeability with the potency correlating to the seriousness of cardiotoxicity. Önyüksel et al. [22] reported that bupivacaine concentration-dependently increased at 100–400 *μ*M the release of carboxyfluorescein from 7.5 mol% cardiolipin-containing liposomes composed of egg yolk phosphatidylcholine and cholesterol, although it showed no significant effects on the membrane permeability of liposomes devoid of cardiolipin. Tsuchiya et al. [23] found that bupivacaine, ropivacaine, lidocaine, and prilocaine increase at 10–300 *μ*M the fluidity of biomimetic membranes with the potency being cardiolipin- ≫ POPA- > POPG- > POPS-containing membranes and that their membrane effects were increased with elevating the anionic phospholipid content in membranes. They also revealed that local anesthetics interact with biomimetic membranes containing 2.5–12.5 mol% cardiolipin to show the rank order of membrane fluidization being bupivacaine ≫ ropivacaine > lidocaine > prilocaine, which agrees with their relative cardiotoxicity.

Since local anesthetics predominantly exist as positively charged molecules at physiological pH, they should electrostatically interact with anionic phospholipids. With respect to the polar head structure, DOPG and POPA have one phosphate group, whereas cardiolipin has two phosphate groups. In the comparative study of anionic phospholipid membranes [23], the relative DPH polarization change induced by bupivacaine was 1.00, 0.40, and 0.49 for 10 mol% cardiolipin-, 10 mol% POPG-, and 10 mol% POPA-containing membranes, respectively, which is consistent with the structural constitution of a cardiolipin polar head group. Cardiolipin is localized in the mitochondrial membranes of cardiomyocytes to play an important role in heart functions, energy metabolism, and membrane dynamics [54]. A relation is presumed between the interaction with mitochondrial membrane cardiolipin and the induction of cardiotoxic effects.

Groban et al. [24] reported that when open-chest dogs were received the incremental infusions of local anesthetics, free and total plasma concentrations at the cardiovascular collapse were 10–38 and 39–100 *μ*M for bupivacaine, 36–142 and 64–163 *μ*M for ropivacaine, and 162–754 and 276–847 *μ*M for lidocaine. While dosing dogs with bupivacaine 7.2–8.8 mg/kg caused cardiac arrhythmia, the plasma concentrations of free and total bupivacaine reached 4 and 17 *μ*M, respectively [25]. In the range of cardiotoxic concentrations, bupivacaine interacts with 2.5–12.5 mol% cardiolipin-containing membranes more intensively and induce the greater membrane fluidization compared with other local anesthetics [23].

Bupivacaine is more hydrophobic than ropivacaine, lidocaine, and prilocaine [18]. The high hydrophobicity of bupivacaine is linked to its strong attraction to myocardial sodium channels, slow dissociation from sodium channels, and access to mitochondrial membranes [55]. The rank order of interactivity with cardiolipin-containing membranes is also correlated to that of cardiotoxicity being bupivacaine > ropivacaine > lidocaine > prilocaine. Since bupivacaine can readily reach mitochondrial membranes even when applied on the outside of cells, the interaction with cardiomyocyte mitochondrial membranes would be responsible for the structure-specific cardiotoxic effects of local anesthetics. A crucial role of anionic phospholipids like cardiolipin in the interaction between local anesthetics and membrane lipids may also lead to the therapeutic possibility for local anesthetic cardiotoxicity [56] as described in Section 7.3.

### 7.3. Treatment of Cardiotoxicity with Lipid Emulsion

Although newly introduced ropivacaine and levobupivacaine are less toxic, they still cause the life-threatening events, so the cardiotoxicity of long-acting local anesthetics remains an important problem. The property of local anesthetics to hydrophobically interact with or selectively bind to membrane lipids may provide a novel strategy for treating their cardiotoxicity. Weinberg et al. [57] reported that the treatment with a lipid infusion shifted the dose response to bupivacaine-induced asystole of rats and dogs to increase the bupivacaine lethal doses. Since then, the rapid infusion of lipid emulsions has been performed to treat the local anesthetic systemic toxicity in animal models and humans [58–60]. The successful resuscitation of local anesthetic-induced cardiovascular collapse was achieved by an intravenous lipid infusion [61], whereas the failure of a lipid emulsion to reverse local anesthetic-induced neurotoxicity is found in the literature [62].

For entrapping cardiotoxic drug molecules, the commonly used lipid emulsions are composed mainly of soy bean and egg phospholipids with triglycerides of varying chain lengths. There are several lipid emulsions available such as Intralipid, Medialipide, Structolipid, and so forth [63]. Although many case reports support the use of an intravenous lipid infusion for the cardiotoxicity of bupivacaine, levobupivacaine, and ropivacaine, the mode of action of this treatment is not fully apparent. One of proposed mechanisms is the “lipid sink” theory, in which the more hydrophobic (lipid soluble) are drug molecules, the greater are their bindings to lipid emulsions, enabling the emulsions to act like a sink that drains cardiotoxic local anesthetics from plasma [60, 63]. This theory is supported by the comparative emulsification degrees of bupivacaine, levobupivacaine, and ropivacaine in the decreasing order, which correlate to the cardiotoxic intensities [64]. The Association of Anesthetists of Great Britain and Ireland released the guidelines for using a lipid rescue therapy [65].

### 7.4. Cardiotoxicity Enhanced by Ischemia

Ischemia potentiates the cardiotoxic effects of local anesthetics and hastens the onset of fibrillation induced by local anesthetics. The electrical ventricular fibrillation thresholds of bupivacaine and ropivacaine were decreased during myocardial ischemia [66]. Pacini et al. [67] revealed that the effect of lidocaine on sheep Purkinje fibers is greater under simulated ischemia than that in normal conditions. Freysz et al. [68] also reported that the risk of cardiac disorders in bupivacaine anesthesia was increased by ischemia. However, the detailed mechanism remains unclear for the local anesthetic cardiotoxicity altered by ischemia. Myocardial ischemia is characterized by a significant lowering of tissue pH to 6.5 or less [69]. Since local anesthetics show greater cardiotoxic, not anesthetic, effects in the presence of acidosis [70], their enhanced cardiotoxicity may be related to ischemic acidic conditions. While local anesthetics block sodium channels to decrease the excitability of myocardia and cause the cardiac malfunction, it has been controversial whether their blocking potency is reduced or enhanced at acidic pH. Tan and Saint [4] reported that the block of cardiac sodium channels by lidocaine was decreased by lowering the pH.

In the comparative study of Tsuchiya et al. [71], bupivacaine and lidocaine of cardiotoxically relevant concentrations interacted with liposomal membranes consisting of 100 mol% DPPC and peripheral nerve cell-mimetic membranes to increase the membrane fluidity, and their membrane effects were decreased by lowering the pH from 7.4 to 5.9. In contrast, the fluidizing effects of bupivacaine and lidocaine on mitochondria-mimetic membranes were reversely increased at pH 5.9–6.4 compared with those at pH 7.4. Such increases under acidic conditions were greater in the biomimetic membranes that contained the substantial amounts of cardiolipin and phosphatidylserine. Positively charged bupivacaine and lidocaine are presumed to interact with the negatively charged head groups of phospholipids, thereby increasing their membrane effects at acidic pH. The interactivity with cardiolipin-containing mitochondrial membranes, which is enhanced by lowering the pH, is associated with the local anesthetic cardiotoxicity enhanced by cardiac ischemia-relating acidosis.

Besides ischemic acidosis, reactive oxygen species [72] and mitochondrial membrane cardiolipin [54] may be also responsible for enhancing local anesthetic cardiotoxicity. Ischemic and reperfused myocardia produce nitric oxide simultaneously with generating superoxide anion, both of which react to form peroxynitrite that pathologically contributes to the myocardial ischemia-reperfusion injury by peroxidizing membrane lipids [73]. The peroxidation of membrane lipids is accompanied by a change in fluidity of liposomal and biological membranes. Cardiolipin constitutes a significant portion of total mitochondrial phospholipids in mammalian cardiomyocytes and plays an important physiological role for hearts. Since hydrophobic local anesthetics readily reach mitochondrial membranes even when applied extracellularly, they can interact with membrane cardiolipin.

Tsuchiya et al. [74] treated biomimetic model membranes of varying lipid compositions with peroxynitrite and local anesthetics separately or in combination. They found that peroxynitrite reacts with the membranes to decrease the membrane fluidity with the potency being cardiolipin- > SAPC- > DPPC-containing membranes. Biomimetic membranes treated with 0.1–10 *μ*M peroxynitrite were more rigid by increasing the membrane cardiolipin content from 0 to 30 mol%, suggesting that cardiolipin is a possible target for peroxynitrite. Cardiolipin in cardiomyocyte mitochondrial membranes preferentially reacts with peroxynitrite [75]. While the peroxidizability of membrane lipids is enhanced as a function of the number of double bonds in lipid molecules, cardiolipin in mammalian cells predominantly contains linoleic acid (C18 : 2) as a side-chain fatty acid [76]. Linoleic acid constitutes 80–90% acyl chains of cardiolipin and tetralinoleoyl cardiolipin is the most common molecular species in cardiomyocyte mitochondrial membranes. Because of its high unsaturation degree, cardiolipin is more susceptible to peroxynitrite compared with other membrane-constituting unsaturated phospholipids. Bupivacaine and lidocaine of each 200 *μ*M were revealed to exert the greater fluidizing effects on biomimetic membranes containing 10 mol% cardiolipin by pretreating the membranes with 0.1 and 1 *µ*M peroxynitrite [74], which is attributable to the membrane rigidity enhanced by peroxynitrite. The drug and membrane interaction is essentially influenced by the inherent fluidity of membranes as bupivacaine and ropivacaine are more effective in fluidizing the membranes that contain a certain amount of membrane-rigidifying cholesterol [77]. Local anesthetics have the property to exert greater membrane-fluidizing effects on the relatively rigid (less fluid) membranes than those on the relatively fluid (less rigid) ones. Peroxynitrite preferentially decreases the fluidity of cardiolipin-containing biomembranes and the resulting membranes are susceptible to the interaction with local anesthetics, which may be partly associated with the local anesthetic cardiotoxicity enhanced by myocardial ischemia.

One may interpret that the membrane effects of bupivacaine and lidocaine lead to the protection against myocardial ischemic insults, not the cardiotoxicity. The cardioprotective effects of local anesthetics were recently revealed to be mediated by their antioxidant and antiapoptotic activities [78, 79]. Although membrane-fluidizing bupivacaine and lidocaine can counteract the membrane rigidification by peroxynitrite, their membrane interactivities are not correlated to such activities [44, 79]. Membrane lipids are completely peroxidized in a very short time by peroxynitrite, resulting in the structural and functional changes of membrane-constituting cardiolipin [80]. The interaction of local anesthetics with peroxynitrite-treated membranes seems to relate to their cardiotoxic effects, not cardioprotective ones. The membrane interactivity of lidocaine was enhanced when mitochondria-mimetic membranes containing cardiolipin were pretreated with ischemic peroxynitrite, but reduced when nerve cell-mimetic membranes not containing cardiolipin were treated with inflammatory peroxynitrite [46], suggesting that the presence or absence of cardiolipin determines whether the membrane interaction of local anesthetics is increased or decreased.

Myocardial ischemia decreases the content of cardiolipin in mitochondria. The 20–25% depletion was indicated for cardiolipin in rabbit heart subsarcolemmal mitochondria [81]. The effects of local anesthetics on membrane fluidity and permeability are influenced by the composition of cardiolipin in membranes [22, 23]. The responsibility of the membrane interaction for local anesthetic cardiotoxicity may be influenced to some degree by varying cardiolipin membrane content.

## 8. Discrimination between Stereoisomers

### 8.1. Local Anesthetic Stereoisomers

More than 50% of currently used drugs are composed of chiral compounds, about 90% of which have been clinically administered as a racemate (racemic mixture) consisting of an equimolar mixture of enantiomers [82]. The chirality of drug molecules generally arises due to the presence of an asymmetric carbon. Enantiomers are a pair of stereoisomers that are mirror images of each other and not superimposable, called chiral.

**Figure 5:**
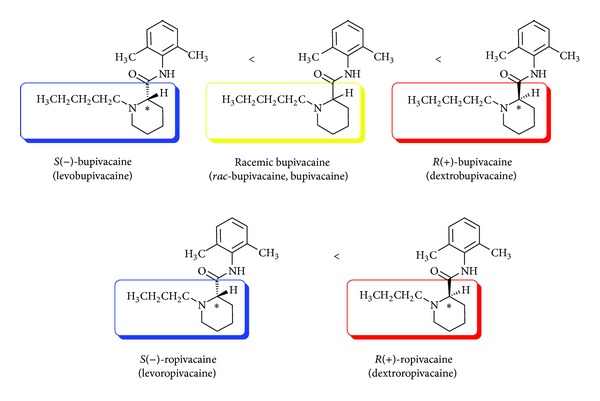
Local anesthetic stereoisomers and their relative cardiotoxicity.

Drug enantiomers are discriminable in qualitative and quantitative pharmacology. Typically, one enantiomer is more active or more toxic than its enantiomeric counterpart, antipode. Stereoselectivity (selectivity to one stereoisomer) is linked to the clinical advantage of using a single enantiomer over its antipode and racemate, which increases the beneficial effect and decreases the adverse effect. Since the clinical use in the 1960s, bupivacaine had been widely marketed as a racemic mixture of bupivacaine (*rac*-bupivacaine) that consists of equimolar enantiomers: *S*(−)-bupivacaine of the levorotatory configuration and *R*(+)-bupivacaine of the dextrorotatory configuration (Figure 5). In the 1990s, however, an urgent problem of its cardiotoxic effects led to the first development of an *S*(−)-enantiomeric local anesthetic, less toxic ropivacaine [83]. This trend was followed by the introduction of levobupivacaine into clinical practice as a pure *S*(−)-enantiomer of bupivacaine [84].

Vladimirov et al. [85] indicated the inhibition ratio of rat neuronal channels to be 1 : 1.3–3 for *S*(−)-bupivacaine and *R*(+)-bupivacaine. Lyons et al. [86] also reported that the relative analgesic effect of *S*(−)-bupivacaine to *rac*-bupivacaine was 0.87 for epidural pain relief in labor. Lim et al. [87] found the stereoselectivity that the analgesic effect of *rac*-bupivacaine is greater than *S*(−)-bupivacaine for patients in labor, while Vladimirov et al. [85] showed that the in vivo nerve block by bupivacaine was less enantioselective (selective to one enantiomer) at clinically used concentrations.

Compared with the analgesic potency, local anesthetics show a greater difference in cardiotoxicity between stereoisomers [51]. Graf et al. [88] found that an *R*(+)-enantiomer prolongs the atrioventricular conduction time more significantly than a racemate and an *S*(−)-enantiomer in isolated guinea pig hearts perfused with 0.5–10 *μ*M bupivacaine stereoisomers. The relative atrioventricular time was 1.54 for *R*(+)-bupivacaine and 1.30 for *rac*-bupivacaine versus 1.00 for *S*(−)-bupivacaine in the perfusion of each 10 *μ*M. Groban et al. [24] carried out the incremental escalating infusions of local anesthetics on open-chest dogs to the point of cardiovascular collapse. Consequently, they found that the mortality is lidocaine, ropivacaine, *S*(−)-bupivacaine, and *rac*-bupivacaine in the increasing order and that the comparative cardiotoxicity is 1.7 for *rac*-bupivacaine versus 1.0 for *S*(−)-bupivacaine. Morrison et al. [89] determined the electrocardiographic cardiotoxic effects of bupivacaine stereoisomers on swine injected with increasing anesthetic doses and showed that the relative median lethal dose was 1.87 for *S*(−)-bupivacaine versus 1.00 for *rac*-bupivacaine.

The major mode of action of local anesthetics is referred to as the block of sodium, potassium, and calcium channels in the nervous and cardiovascular system. Valenzuela et al. [90] found that bupivacaine blocks the relevant sodium channels so enantioselectively that *R*(+)-enantiomer is 1.2–1.7 times more potent than *S*(−)-enantiomer in isolated guinea pig ventricular myocytes. Valenzuela et al. [91] also reported that the relative potency to block cloned human cardiac potassium channels was 1.6 for *R*(+)-bupivacaine versus 1.0 for *S*(−)-bupivacaine. Pharmacological studies have been exclusively focusing on the interactions of local anesthetics with proteinous ion channels, receptors, and enzymes. This is mainly due to the stereostructure-specific actions on functional proteins that can be understood by the enantioselective affinity of drugs for proteins [83]. However, the protein-interacting theory does not fully explain the pharmacodynamic difference between bupivacaine stereoisomers, requiring an alternative or additional explanation for the discriminable effects of local anesthetic stereoisomers.

### 8.2. Enantioselective Membrane Interactivity

Tsuchiya and Mizogami [20] reported that local anesthetic stereoisomers increased the fluidity of nerve cell-mimetic membranes with the potency being* S*(−)-enantiomer < racemate < *R*(+)-enantiomer and also the fluidity of cardiomyocyte-mimetic membranes with the potency being *S*(−)-ropivacaine < *S*(−)-bupivacaine < *R*(+)-bupivacaine. However, these stereoisomers were not discriminated in interactivity with the membranes devoid of cholesterol. When 40 mol% and more cholesterol were contained in membranes, the membrane interactivity was *S*(−)-bupivacaine < *rac*-bupivacaine < *R*(+)-bupivacaine and *S*(−)-ropivacaine < *R*(+)-ropivacaine, which agreed with the rank order of their cardiotoxicity [77]. Mizogami et al. [92] also reported that bupivacaine stereostructure-specifically interacted with biomimetic membranes containing cholesterol to show the relative potency consistent with the clinical features of bupivacaine stereoisomers. Membrane cholesterol is essential to the enantiomeric discrimination of local anesthetics.

The membrane lipid-interacting theory is still controversial as to whether local anesthetics actually act on biomembranes at cardiotoxically relevant concentrations. At 5–200 *μ*M covering the free plasma concentrations for bupivacaine stereoisomers to cause cardiovascular collapse [24], Tsuchiya and Mizogami [93] proved that bupivacaine stereoisomers are able to modify the fluidity of cardiomyocyte-mimetic membranes even at low micromolar concentrations with the potency being *S*(−)-bupivacaine < *rac*-bupivacaine < *R*(+)-bupivacaine. While the bupivacaine-induced changes in DPH fluorescence polarization are considered relevant to the clinical effects [23], the difference between stereoisomers varied depending on their tested concentrations [93], which may account for the inconsistent results of previous studies. The comparative effects of *R*(+)-bupivacaine, *rac*-bupivacaine, and *S*(−)-bupivacaine to produce arrhythmia and cardiovascular collapse differed in experimental and clinical studies [24, 51, 89]. An analgesic effect of *rac*-bupivacaine was greater than that of *S*(−)-bupivacaine in humans [87], whereas bupivacaine was much less enantioselective in the in vivo rat nerve block at clinically used concentrations [85]. When comparing the blocking effects on human nerves, *S*(−)-bupivacaine showed an almost similar potency to *rac*-bupivacaine [84]. These inconsistencies are attributable to the concentration-dependent enantiomeric discrimination that the difference in membrane interactivity between *R*(+)-bupivacaine and *S*(−)-bupivacaine steeply increases at concentrations lower than 50 *μ*M [93].

### 8.3. Chirality of Lipid Membranes

Because drug enantiomers absolutely differ in spatial configuration, they should behave differently when interacting with chiral systems, while not being in achiral environments such as an aqueous solution. In fact, enantiomeric compounds of each other show the different interaction with other compounds that are also enantiomers. Almost all of substances in the body are composed of enantiomeric biocompounds, therefore chiral drugs could interact enantioselectively with such chiral macromolecular targets. The conventional protein-interacting theory emphasizes the importance of proteinous acting sites because proteins are entirely made up of L-amino acids, not D-amino acids. Drug enantiomers form the different spatial relationship in the asymmetric environments of functional proteins that are composed of only L-amino acids. However, the action of enantiomeric local anesthetics on ion channels is not necessarily consistent with the stereoselectivity that an *R*(+)-enantiomer is more potent than an *S*(−)-enantiomer. Previous comparisons showed that *S*(−)-bupivacaine blocked ion channels with the potency equal to or greater than *rac*-bupivacaine [94, 95].

Considering that amphiphilic drugs pharmacodynamically and pharmacokinetically interact with lipid bilayers, it is reasonable to presume that membrane lipids could play a significant role in the in vivo discriminative recognition of such drug molecules. Local anesthetics penetrate into membrane lipid bilayers, align between phospholipid acyl chains, and occupy the space to perturb the phospholipid acyl chain alignment. If local anesthetics stereostructure-specifically interact with chiral components in lipid bilayers, they would modify membrane fluidity with the potency being different between stereoisomers. Tsuchiya and Mizogami [96] hypothesized that drugs interact enantioselectively with chiral lipid membranes to induce membrane fluidity, order, and permeability changes that were discriminable between drug enantiomers. The enantioselective lipid environments to discriminate stereoisomers may be provided by phospholipids and/or cholesterol with the chirality.

The enantiomeric discrimination arises from the direct interaction with chiral centers. Based on the hypothesis that a chiral carbon in their glycerol backbone may allow phospholipids to behave as a chiral component in membranes, phospholipids were speculated to interact preferentially with molecules having the same chirality and exhibit the selectivity to one enantiomer over its counterpart [97]. However, any membrane phospholipids were not found to produce the discrimination of local anesthetic *S*(−)-enantiomers from their antipodes [77]. Compared with phospholipids, cholesterol has much more chiral carbons. It is very likely that cholesterol enhances the chirality of lipid bilayers and its absolute configuration modulates the membrane stereoselectivity [98].

In comparisons of the membrane interactivity between bupivacaine stereoisomers [20, 77, 93], the used probe DPH partitions into the center of lipid bilayers [92], the membrane component cholesterol is located in lipid bilayers with its backbone embedded in the hydrocarbon core [99], and bupivacaine is preferentially localized in the hydrophobic membrane core [19]. Different polarization changes are attributed to the enantioselective interaction of bupivacaine, not to the differential affinity of bupivacaine enantiomers for specific membrane regions. The opposite configurations are considered to allow drug enantiomers to be discriminated by their interaction with another chiral molecule in biomembranes. Membrane cholesterol contributes to such an enantioselective interaction by increasing the chirality of lipid bilayers. The stereostructure and membrane interactivity relationship supports the clinical use of *S*(−)-enantiomers to decrease the adverse effects of local anesthetics on the cardiovascular system.

## 9. Interaction Preference for Membrane Microdomain Lipid Rafts

In the late 1990s, the fluid mosaic model of Singer and Nicholson for biomembranes was evolved to a more sophisticated concept, especially concerning the membrane lipid composition and molecular organization. It has become clear that biomembranes are not the simple bilayer structure with uniformly distributed lipids but organized into phase-separated microdomains, called lipid rafts, with specific lipid components and molecular dynamics that differ from the surrounding liquid crystalline phase and bulk membranes [100]. In lipid rafts, cholesterol and sphingolipids are packed in a highly ordered structure (liquid ordered) distinct from the rest of membranes (liquid disordered). There is accumulating evidence to indicate that lipid rafts are the target of various drugs including general anesthetic agents [101]. However, the interaction of local anesthetics with membrane microdomain lipid rafts is unclear.

Ion channels, *β*-adrenergic receptors, and signaling proteins were discovered to be localized to membrane microdomains within the cardiovascular system [102, 103]. Drugs and chemicals were also revealed to initiate and facilitate their effects by interacting with membrane microdomain lipid rafts [104, 105]. As one of possible pharmacological mechanisms, it is of much interest to know whether local anesthetics more intensively or selectively interact with the lipid rafts surrounding ion channels compared with overall lipid bilayers. Kamata et al. [106] recently reported that lidocaine disrupted the raft structures in human erythrocyte membranes. They also reported that lidocaine reversibly prevented the raft formation in human erythrocyte membranes, suggesting the involvement of raft-related signal transduction in the mode of local anesthetic action [107].

In order to verify the hypothesis that local anesthetics could interact preferentially with lipid rafts over nonraft membranes, Tsuchiya et al. [108] compared the effects of 50–200 *μ*M local anesthetics on raft model membranes, cardiolipin-containing biomimetic membranes, and cardiomyocyte mitochondria-mimetic membranes. Local anesthetics interacted with nonraft membranes to increase the membrane fluidity with the potency being *R*(+)-bupivacaine > *rac*-bupivacaine > *S*(−)-bupivacaine > ropivacaine > lidocaine > prilocaine, which is consistent with the rank order of cardiotoxic potency. These local anesthetics also acted on raft-like liquid-ordered membranes, but any raft model membranes showed neither drug structure dependence nor stereoselectivity in membrane interaction, leading to the conclusion that the mechanistic relevance of membrane microdomain lipid rafts to local anesthetics is questionable at least in their effects on raft model membranes. Bandeiras et al. [109] also reported that lidocaine interacted with raft model membranes, although its interactivity was weaker compared with nonraft membranes.

## 10. Anesthetic and Phytochemical Membrane Interaction

### 10.1. Drug Interaction with Phytochemicals

While the concomitant use of medicines with medicinal plants or herbs has been increasing in popularity, it potentially causes the beneficial or adverse drug interaction with plant components. Unlike the drug-drug interaction, however, the interaction between drugs and phytochemicals (bioactive chemical substances in plants) has not been extensively investigated despite the possibility of more frequent occurrence than expected [110]. Various drugs appear to show the antagonistic, additive, and synergistic interactions with phytochemicals [111].

### 10.2. Capsaicin

Topical or systemic lidocaine therapy is expected to provide a novel management of neuropathic pain symptoms together with enhancing the utility by combining with capsaicin [112]. Capsaicin, 8-methyl-*N*-vanillyl-6-nonenamide, is a pungent component of plants belonging to the genus *Capsicum* such as chili pepper. On the initial application, this phytochemical shows an excitatory effect to produce burning pain and hyperalgesia, whereas with the repeated or prolonged application it shows an inhibitory effect on the receptive terminals of nociceptors. Capsaicin activates a transient receptor potential vanilloid (TRPV1) receptor, which belongs to a family of TRP channels, to exert an analgesic or algesic effect depending on its concentrations [113]. Capsaicin-activating TRPV1 receptor is a nonselective cation channel that plays an important role to modulate the nociceptive and pain transmission in the peripheral and central nervous system [114, 115]. Besides the use as a food additive in spicy cuisines, capsaicin is currently used for the therapy to treat painful conditions such as diabetic neuropathy and rheumatoid arthritis.

**Figure 6:**
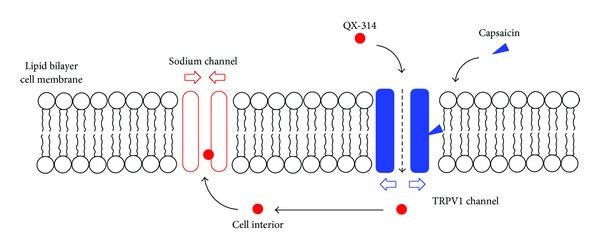
Combined use of QX-314 and capsaicin.

Binshtok et al. [116] suggested that capsaicin could transport a sodium channel blocker QX-314 to nociceptors through the activation of TRPV1 channels and produce the nociceptive-selective analgesia. In their whole-cell voltage-clamp recordings from rat dorsal root ganglion neurons, externally applied QX-314 showed no effects on the sodium channel activity of small sensory neurons when it was applied alone. By applying together with capsaicin, however, QX-314 blocked sodium channels to induce a long-lasting reduction of pain sensitivity. Ries et al. [117] found that a TRPV1 receptor agonist capsaicin accelerates the onset kinetics of QX-314 in a mouse tail-flick test, whereas a TRPV1 receptor antagonist capsazepine decreases the nerve blocking efficacy of QX-314. Permanently charged cationic QX-314 cannot pass through the lipid bilayers of cell membranes but can gain an access to the cell interior through TRPV1 channels that are opened by capsaicin (Figure 6). Once inside, QX-314 is able to bind to the acting site on sodium channels. Shen et al. [118] reported that the peripheral block of nociceptive afferents by QX-314 combined with capsaicin was effective in reducing neuropathic pain. It was also suggested that capsaicin could be applied in combination with bupivacaine and lidocaine instead of QX-314 [119]. Because the transmembrane access rout for QX-314 occurs only on nociceptors, the combination of QX-314 or local anesthetics with capsaicin is expected to produce the ideal analgesia affecting pain alone or the pain-restricted local anesthesia preserving motor and autonomic responses [120]. Shin et al. [121, 122] found that capsaicin significantly potentiates at 50 *μ*M the rat sciatic nerve block by lidocaine. The cooperative drug interaction with capsaicin also would be useful for increasing and prolonging local anesthetic effects.

Capsaicin acts on lipid bilayers to modify the membrane fluidity as well as local anesthetics and QX-314 [123]. Experimental data show that capsaicin interacts with nerve cell-mimetic membranes to change the membrane fluidity and that the combined use with 50 *μ*M capsaicin additively increases the membrane effects of lidocaine and bupivacaine of clinically relevant concentrations [124]. Capsaicin may influence the effects of local anesthetics by cooperatively enhancing their interactivity with biomembranes.

### 10.3. Phloretin

Flavonoid phloretin is a polyphenol primarily contained in apples and their products, which has been suggested to possess the antioxidant and cancer-preventive activity [125]. Tsuchiya and Mizogami [124] reported the membrane interaction between phloretin and local anesthetics. They treated biomimetic model membranes with lidocaine, bupivacaine, or phloretin alone and in combination and then determined the membrane fluidity changes by measuring DPH fluorescence polarization. Consequently, phloretin was revealed to act on nerve cell-mimetic membranes and decrease the membrane fluidity in contrast to local anesthetics increasing the membrane fluidity. The combined use with 25 *μ*M phloretin antagonistically inhibited the membrane effects of lidocaine and bupivacaine of clinically relevant concentrations. Phloretin was also reported to affect the membrane structure and the local anesthetic permeability of lipid monolayers and bilayers [126, 127].

A phloretin derivative (polymeric mixture of polyesters of phloretin and phosphoric acid) was found to shorten the duration time of infiltration anesthesia of guinea pigs by lidocaine, bupivacaine, and prilocaine and to terminate their local anesthetic effects [128]. Its local anesthetic-inhibitory effect was also proved in the infiltration anesthesia of human teeth [129]. As a clinical utility of the drug and phytochemical interaction, phloretin may be valuable to discontinue local anesthesia as soon as the treatment is completed.

## 11. Conclusions

Membrane-embedded ion channels, especially sodium channels, have been recognized to be primarily responsible for the pharmacologic and toxic effects of local anesthetics. However, the drug and lipid membrane interaction is not disregardable as the mode of local anesthetic action. While channel proteins have the affinity specific to individual ligand structures, they cannot necessarily interact with all of structurally different drugs. Membrane lipids also play an important role in the differential recognition of drug structures. A substantial number of administered local anesthetic molecules must be transported to the target through the lipid barriers of nerve sheaths and penetrate into or across the lipid bilayers of cell membranes. Amphiphilic local anesthetics hydrophobically and electrostatically interact with lipid bilayers to modify the fluidity of biomembranes with the potency correlating to the anesthetic activity and the cardiac toxicity. Such interactions result in not only the direct influence on membrane functions but also the influence on channel functions by changing the conformation of transmembrane proteins. Membrane components, phospholipids, and cholesterol, rich in molecular species and chirality, allow the membrane interaction of local anesthetics to be structure dependent and stereostructure selective.

The interaction between local anesthetics and membrane lipids would be associated with both the basic mechanisms for anesthetic and cardiotoxic effects and the clinical features such as the activity and toxicity altered by pathophysiological conditions, the cardiotoxicity varying by structural differences and discriminating between stereoisomers, the utility of a lipid emulsion to treat cardiotoxicity, and the combination with phytochemicals. The interaction with only functional proteins cannot explain all of the pharmacological and toxicological characteristics of local anesthetics nor can the interaction with only membrane lipids. Membrane lipid-interacting theory and membrane protein-interacting theory are complimentary to each other, suggesting the modified lipid-protein interaction mechanism for local anesthetics.

## Abbreviations

DPH: 1,6-Diphenyl-1,3,5-hexatriene
DPPC: 1,2-Dipalmitoyl-*sn*-glycero-3-phosphocholine
*n*-AS: *n*-(9-Anthroyloxy)stearic acid (*n* = 2, 6, 9 and 12)
PNA: *N*-Phenyl-1-naphthylamine
POPA: 1-Palmitoyl-2-oleoyl-*sn*-glycero-3-phosphate
POPC: 1-Palmitoyl-2-oleoyl-*sn*-glycero-3-phosphocholine
POPE: 1-Palmitoyl-2-oleoyl-*sn*-glycero-3-phosphoethanolamine
POPG: 1-Palmitoyl-2-oleoyl-*sn*-glycero-3-[phospho-*rac*-(1-glycerol)]
POPI: 1-Palmitoyl-2-oleoyl-*sn*-glycero-3-phospho-(1′-myo-inositol)
POPS: 1-Palmitoyl-2-oleoyl-*sn*-glycero-3-[phospho-L-serine]
QX-314: *N*-Ethyl lidocaine
SM: Sphingomyelin
TMA-DPH: 1-(4-Trimethylammoniumphenyl)-6-phenyl-1,3,5-hexatriene
TRPV1 receptor: Transient receptor potential vanilloid receptor.
